# Trends in the Prevalence of Tuberous Sclerosis Complex Manifestations: An Epidemiological Study of 166 Japanese Patients

**DOI:** 10.1371/journal.pone.0063910

**Published:** 2013-05-17

**Authors:** Mari Wataya-Kaneda, Mari Tanaka, Toshimitsu Hamasaki, Ichiro Katayama

**Affiliations:** 1 Department of Dermatology, Graduate School of Medicine, Osaka University, Suita, Osaka, Japan; 2 Department of Biomedical Statistics, Graduate School of Medicine, Osaka University, Suita, Osaka, Japan; Vanderbilt University Medical Center, United States of America

## Abstract

Tuberous sclerosis complex (TSC) is an autosomal dominant disorder with multi-system involvement and variable manifestations. There has been significant progress in TSC research and the development of technologies used to diagnose this disorder. As a result, individuals with mild TSC are now being diagnosed, including many older adults who have not developed seizures or cognitive abnormalities. We conducted a statistical analysis of the frequency of TSC manifestations in a population of Japanese adults and children, comparing our findings with historical data. The chi-square test was used to examine the frequency of each manifestation by age. A total of 166 outpatients at the Department of Dermatology of Osaka University Hospital during the period from January 2001 to March 2011 were included in the study. Compared to previous reports, the frequency of neurologic manifestations (excepting autism) was lower in this cohort, and the frequency of skin manifestations (excepting hypomelanotic macules) was higher in this cohort. The frequencies of pulmonary lymphangioleiomyomatosis and renal manifestations were not significantly different from those previously reported. Regarding the association of each manifestation with age, the frequency of neurologic manifestations (excepting subependymal giant cell astrocytoma) was significantly higher in younger patients than in older patients. The frequency of skin manifestations and renal angiomyolipoma were significantly higher in older patients than in younger patients. Because of their high frequency and visibility, skin manifestations are useful in the diagnosis of TSC. Moreover, uterine perivascular epithelioid cell tumor was also characterized as a new findings associated with TSC.

## Introduction

Tuberous sclerosis complex (TSC) is an autosomal dominant genetic disorder with a birth incidence of 1∶6000 [1]. TSC can affect nearly every organ system, with various manifestations occurring at various times throughout the individual’s lifetime.

Classically, TSC was identified by the triad of facial angiofibromas, intellectual disability, and epilepsy. Two responsible genes, *TSC1* [2] and *TSC2* [3], which encode hamartin and tuberin, respectively, were discovered in the 1990s, improving the diagnosis of TSC. Mildly affected individuals are now being diagnosed, including many older adults who do not have seizures or intellectual disabilities. This evolution of diagnostic testing has resulted in changes in the prevalence of each manifestation of TSC over the past two decades. Genetic testing has recently become available, suggesting relationships among subependymal nodule (SEN), epilepsy [4], intellectual disability [5], forehead plaque, renal angiomyolipoma [6] and *TSC2* genotypes [7], [8], [9]; however, many factors modify individual phenotypes, and the use of genotyping to predict the manifestations of TSC in individuals is difficult. Furthermore, 10–15% of TSC patients do not have identified mutations in *TSC1* and *2* [5], [9]. Thus, a precise analysis of the recent epidemiological data concerning TSC manifestations is necessary. Although there have been reports on the epidemiology of TSC [10], [11], [12], [13], [14], most of these reports focus on younger subjects, with few studies including patients 20 years of age and older. TSC is a heterogeneous disease with a highly variable clinical presentation, which also varies with the age of the patients. The prevalence of each manifestation differs depending on the age composition of the study population. Therefore, an epidemiologic survey including both adults and children is required to understand the current manifestations and natural history of the disease; these results will support the appropriate management of TSC patients.

In this study, we analyzed the frequency and characteristics of the clinical symptoms of 166 Japanese TSC patients treated in the Department of Dermatology, Osaka University Hospital, including patients 20 years of age and older, to elucidate the current trends in TSC manifestations. The aim of this study was to elucidate the epidemiology of TSC manifestations in Japan and to examine the prevalence of each manifestation by age.

## Methods

### Study Population

A total of 166 outpatients treated in the Department of Dermatology of Osaka University Hospital during the period from January 2001 to March 2011 were enrolled. The subjects were diagnosed with TSC according to Roach’s clinical diagnostic criteria, which were devised in 1998 at the Tuberous Sclerosis Complex Consensus Conference in Annapolis, Maryland, USA [15]. Patients with probable TSC were excluded. Upon their enrollment in the study, the most recent data for all the patients were recorded. Although the 166 patients visited our hospital from all over Japan and had various types of TSC, as no public health data about Japanese TSC patients was available, it is not known whether these data are representative of TSC patients in Japan.

### Manifestations

The neurological clinical manifestations analyzed included subependymal giant cell astrocytoma (SEGA), subependymal nodule (SEN), epilepsy and neurodevelopmental psychiatric/cognitive symptoms. SEN and SEGA were identified in the TSC patients using brain magnetic resonance imaging (MRI) and computed tomography (CT). The presence of SEGA indicates a growing SEN with a diameter greater than 10 mm. Three levels of epilepsy were identified: no seizures, seizures without refractory epilepsy (slight epilepsy) and refractory epilepsy. Refractory epilepsy was defined as the occurrence of uncontrolled seizures at least twice per week despite the administration of more than two different anti-epileptic drugs. The neurodevelopmental and psychiatric/cognitive symptoms were difficult to assess precisely because they comprise many issues, each of which influences the others; therefore, we focused on only two issues: autism/ASD and intellectual disability (mental retardation). Autism and ASD were diagnosed in specialized facilities in pediatric or psychiatric departments, as there is no diagnostic criteria-based consensus in Japan. Intellectual functioning was divided into four grades: normal and levels 1 to 3. Level 1 functioning indicates that a social life is possible. Level 2 indicates that patients can conduct their daily lives without assistance, and level 3 indicates that care is required in daily life.

The renal manifestations of TSC include angiomyolipomas (AML), renal cysts and, rarely, carcinomas. CT, MRI or ultrasonography (US) were used to examine 153 of the 166 patients. Of the 13 patients who were not examined, 11 (85%) were younger than 20 years of age.

Lymphangioleiomyomatosis (LAM) and multifocal micronodular pneumocyte hyperplasia (MMPH) are the pulmonary manifestations of TSC. The diagnosis of LAM and MMPH depends on the use of high-resolution CT (HRCT). Of the 166 TSC patients, 95 individuals were examined using HRCT. The 58 individuals (82%) who were not examined using this method were younger than 19 years of age. Of the 95 examined patients, 67 (71%) were female, and 28 (29%) were male.

The observed skin manifestations included facial angiofibromas (AFs), forehead plaques, hypomelanotic macules, shagreen patches and ungual fibromas. The AFs were divided into three classes: slight, mild and severe. Slight AF indicates that the AFs numbered less than 50, and all of the lesions were solitary and not fused. In addition, each tumor had a diameter smaller than 2 mm or telangiectasis. Severe AF indicates that the total area of the lesions covered more than two-thirds of the face and that some of the AFs had fused to create plaques or tumors thicker than 10 mm and with a major axis longer than 70 mm. Mild AF indicates that the seriousness of the AFs was between slight and severe. Forehead plaques included facial and scalp plaques. Both types of ungual fibroma, i.e., periungual and subungual fibromas, were included. A longitudinal groove without a visible fibroma was also classified as an ungual fibroma.

Cardiac manifestations were investigated using echocardiography and electrocardiography in 106 patients (45 males and 61 females). All patients with histories of cardiac rhabdomyoma were examined, although the majority of these patients had no current symptoms.

The uterus was examined in 51 of 66 female patients over 20 years of age using abdominal CT and MRI. Three patients had already undergone radical hysterectomies because of multiple uterine leiomyomas, perivascular epithelioid cell tumor (PEComa) and carcinomas. The ovaries were examined in 39 female patients over 20 years of age using abdominal ultrasonography, MRI and CT.

Low-density areas on the thyroid gland were often observed during investigations of the patients’ pulmonary prognosis via thoracic CT. The 26 patients who had this abnormality were thoroughly examined using ultrasonography of the thyroid gland and histological analyses of needle-biopsy specimens.

### Genetic Analysis

Genomic DNA was extracted from whole blood using a Sepa Gene Kit (Sanyo Junyaku, Tokyo, Japan). PCR amplification of all exons in the *TSC1* and *TSC2* genes was performed [16] using previously published primers. The PCR products were applied to a denaturing high performance liquid chromatography (DHPLC) machine, a WAVE® Nucleic Acid Fragment Analysis System equipped with a DNASep® cartridge (Transgenomic, Omaha, NE, USA). The PCR products that displayed heteroduplex peaks in the DHPLC chromatogram were then directly sequenced using an ABI Prism® 310 Genetic Analyzer (Applied Biosystems, Foster City, CA, USA). When a deletion or insertion mutation was suspected through direct sequencing analysis, we sequenced subcloned fragments in pGEM T-Easy Vector Systems (Promega, Madison, WI, USA) to identify the mutation.

### Statistics

To examine the trends in each manifestation, the frequencies of each manifestation in our population were statistically compared with data from the recent literature (Table 1). The frequency of each manifestation is presented as a percentage with a 95% confidence interval (CI). The prevalence of each manifestation was compared with recent literature using the chi-square test. The prevalence and severity of each TSC symptom differ depending on the age composition of the study population. Therefore, we also stratified the results by 10-year age groups. The frequency of the manifestations in each age group was also compared using the chi-square test. The variables influencing intellectual disability, refractory epilepsy and autism/autism spectrum disorder (ASD) were compared using the chi-square test. Then, these data were analyzed using a generalized logistic model. A statistical analysis of the age difference between males and females was conducted using the Wilcoxon test. The 95% CI and *p* value were reported for significant predictors. A *p* value <0.05 was considered statistically significant. All statistical analyses were conducted in SAS for Windows 9.3 software (SAS Institute, Cary, NC, USA).

**Table 1:** Frequency of each manifestation compared with historical data.

Manifestations of TSC	Frequency (%)	95% CI	Reference	*p*-value	Result
**Neurological manifestations**					
SENs(including SEGA) [7], [9], [10], [14]	77	70–84	50–100	1.00	ND
SEGA [7], [9], [10], [14], [17]	2	0–5	10–35	<0.0001	L
Epilepsy [4], [7], [8], [9], [10], [18], [19], [20], [21], [22]	63	56–70	75–95	0.0005	L
Refractory epilepsy [4]	20	14–26	50	<0.0001	L
Interectual disability [7], [9], [12], [14], [31], [32], [33]	42	34–49	65–75	<0.0001	L
Level 3	3.6	1–6.			
Level 2	21	15–27			
Level 1	17	11–23.			
Autism [22], [24], [25], [26]	21	15–27	25–50	0.24	ND
**Renal manifestations** [6], [7], [8], [9], [34], [35], [36]	71	64–78	64–78		
Angiomyolipomas [6], [7], [8], [9], [36]	61	53–69	45–55	1.00	ND
>4 cm	29	22–36			
Cysts [6], [7], [8], [9], [36]	28	21–35	15–32	0.65	ND
Renal cell carcinomas [6], [7], [8], [9], [36]	2.6	0–5	2	0.59	ND
**Pulmonary manifestations**	79	71–87			
LAM [14], [39], [40], [41], [42], [43]	39	29–49	2–40	0.92	ND
MMPH	71	62–80			
**Skin lesions**	98.8	97–100			
Facial angiofibromas [7], [14], [45], [46], [48], [49]	93	89–97	75–80	<0.0001	H
Fiorehead plaques [7], [8], [9], [12], [14], [34], [45], [46], [49], [50]	46	38–54	12–40	0.13	ND
Hypomelanotic macules (>3) [44], [46], [47], [48], [51]	65	58–72	>90	<0.0001	L
Shagreen patches [14], [44], [45], [46], [47], [48]	83	77–89	20–57	<0.0001	H
Ungual fibromas [14], [45], [46], [48], [54]	64	57–71	15–80	1.00	ND
**Cardiac manifestations**	49	39–59			
Cardiac rhabdomyomas [7], [8], [9], [12], [14]	46	37–55	40–60	0.90	ND
**Reproductive** (female older than 20)					
Uterine manifestations (>20 years old)	57	43–71			
Ovarian cyst	28	14–42			
**Endocrine organ**					
Thyroid	27	18–36			
**Genotype** [5], [7], [8], [9]					
TSC1	28	18–38	20	0.08	ND
TSC2	32	21–43	66	<0.0001	L
No mutation identified (NMI)	39	30–52	20	<0.0001	H

SEN : subependymal nodule.

SEGA : subependymal giant cell astrocytoma.

LAM : lymphangioleiomyomatosis.

MMPH : multifocal micronodular pneumocyte hyperplasia.

CI : confidence interval.

H : significantly higher.

L : significantly lower.

ND : no significant difference.

### Ethical Considerations

This study was approved by the ethics committee of the Osaka University Faculty of Medicine. Written informed consent was not needed because this study retrospectively collected available medical records in the hospital. The ethics committee of the Osaka University Faculty of Medicine approved this consent procedure.

Genetic analysis was performed by another inspection institution under the approval of the ethics committee of the Osaka University Faculty of Medicine. Written informed consent from the participants or the guardians on the behalf of the minors/children for genetic analysis was obtained at each institution where the analysis was performed. The ethics committee of the Osaka University Faculty of Medicine approved the genetic analysis of TSC patients at other institutions. We were permitted to use the database of the results of genetic analysis by the ethics committee of the Osaka University Faculty of Medicine.

## Results and Discussion

### Patient Characteristics

**Figure 1:**
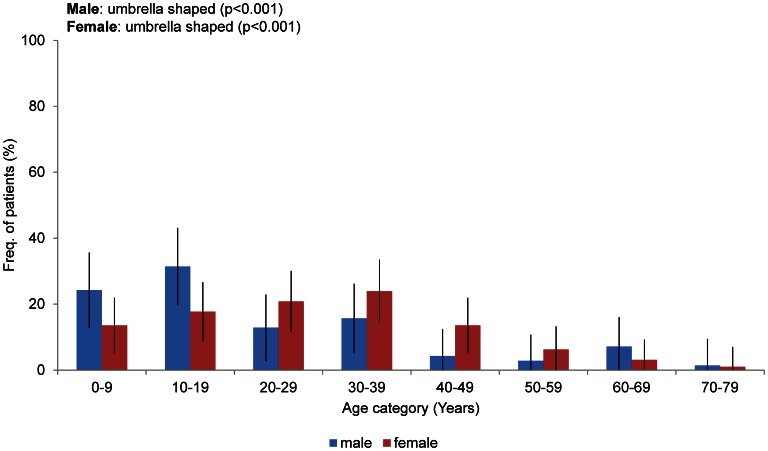
Age and gender of the 166 individuals in the study population. The rates indicate the percentages of patients in each age group. The bars indicate the confidence interval (CI).

There were 70 males and 96 females in the study group. Their ages ranged from 0 to 78 years of age, with a mean of 26.6 years. The mean age of the males was 18.5 years, and the mean age of the females was 28.0 years. The age distribution by decade is shown in Figure 1. Eighty percent of the patients were younger than 50 years of age, and 52% of the patients were older than 20 years of age. Of the 166 individuals (145 families), 36 (10%; 15 families) were familial cases, and 130 (90%) were sporadic cases. The shape of the prevalence with age differs between the male and female patients to a significant degree (p = 0.01). The prevalence peak lies between 10–19 years old for males and 30–39 years old for females.

### Neurological Manifestations

**Figure 2:**
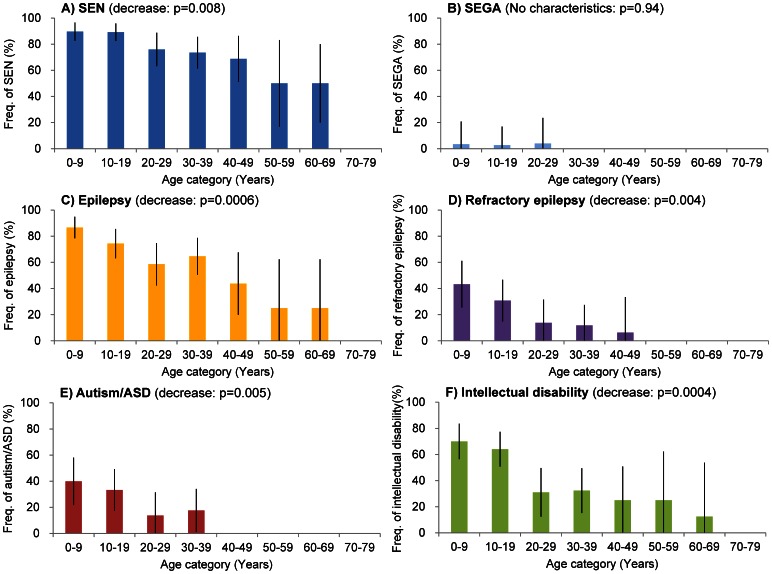
Frequency of neuronal manifestations in each age group. The rates of A) subependymal nodule (SEN: blue), B) subependymal giant cell astrocytoma (SEGA; light blue), C) epilepsy (orange), D) refractory epilepsy (purple), E) autism/ASD (red) and F) intellectual disabilities (green) in each age group are shown. Intellectual disabilities include any degree of disability. SEGA and refractory epilepsy were defined as described in the text. The rates indicate the percentages of patients with each manifestation in each age group. The bars indicate the confidence interval (CI).

The frequency of SEGA (2%) among our patients was significantly lower in this study than in previous reports [7], [9], [10], [14], [17] (Table 1). There was no significant association between SEGA and age (Fig. 2 B). In contrast, the frequency of SEN showed a significant association with age such that as age increases, the occurrence of SEN decreases (Fig. 2A).

Epilepsy has been reported in 75–95% of patients with tuberous sclerosis complex [4], [7], [8], [9], [10], [18], [19], [20], [21], [22], and 62.5% of these epilepsy patients develop refractory epilepsy [4]. As shown in Table 1, 37% of the TSC patients did not have epilepsy, 43% had slight epilepsy and only 20% had refractory epilepsy. The frequency of both epilepsy and refractory epilepsy was significantly lower than in previous reports (Table 1). There was a significant association between epilepsy, refractory epilepsy and age. The frequency of both decreased in the older groups (Fig. 2C, D). There were 54 male patients (77%) and 51 female patients (53%) with epilepsy. The incidence was significantly higher in males (*p* = 0.0159) than in females (*p* = 0.0395).

Autism and ASD are strongly associated with TSC. The prevalence of autism/ASD is 1% in the general population [23] and 25–50% among children with TSC [22], [24], [25], [26]. The frequency of autism/ASD in our patients (21%) was at the lower end of the range observed among children with TSC but higher than that in the general population. There was no significant frequency difference in our cohort compared to the previously reported prevalence (Table1). Autism/ASD was significantly associated with age, and the frequency decreased as age increased (Fig. 2E). The 35 patients with autism/ASD included 20 (29%) males and 15 (16%) females. The frequency of autism/ASD was significantly higher in males than in females (*p* = 0.04).

Intellectual disabilities have traditionally been considered a hallmark of TSC [27], [28], [29], [30], with a high occurrence among these patients, but 58% of the studied patients had no intellectual disabilities. The rates of severe intellectual impairment were low (3.6%). The frequency of intellectual disability observed here, 42%, was significantly lower than in previous reports [7], [9], [12], [14], [31], [32], [33] (Table1). Recently, a low prevalence of intellectual disabilities among TSC patients has also been reported [8], [32]. The frequency of intellectual disabilities decreased significantly with increasing age (Fig. 2F).

**Figure 3:**
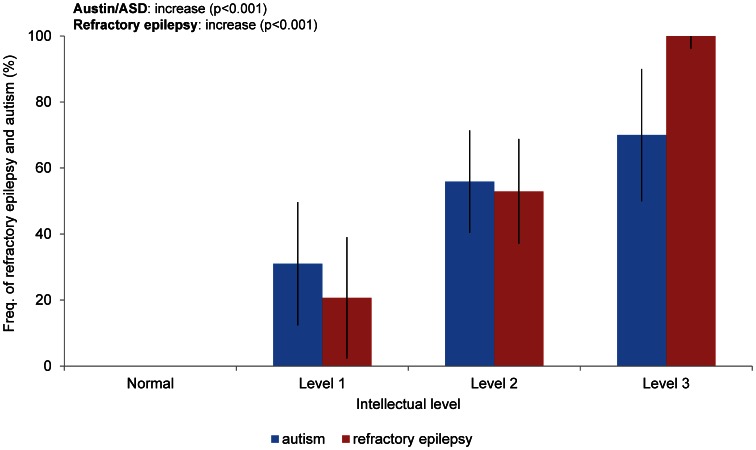
Association of refractory epilepsy and autism/ASD with intellectual disabilities. Frequency (%) of patients with refractory epilepsy (red column) and autism/ASD (blue column) at each intellectual level are shown. The statistical analyses of refractory epilepsy and autism/ASD in relation to intellectual disability were examined using the chi-square test, and the generalized logistic models showed significance at *p*<0.001. The bars indicate the CI.

The three neurologic symptoms of TSC, refractory epilepsy, autism/ASD and intellectual disabilities, were interrelated. The relationship of intellectual disability with refractory epilepsy and autism/ASD is shown in Figure 3. All patients with severe (level 3) intellectual disabilities had refractory epilepsy. In contrast, no patients without intellectual disabilities had refractory epilepsy.

Regarding the relationship between refractory epilepsy, autism/ASD and intellectual disabilities, the rate of refractory epilepsy and autism/ASD increased as the severity of the intellectual disability increased (Fig. 3). The rates of refractory epilepsy and autism/ASD were significantly associated with severe intellectual disabilities (Fig. 3).

### Renal Manifestations

**Figure 4:**
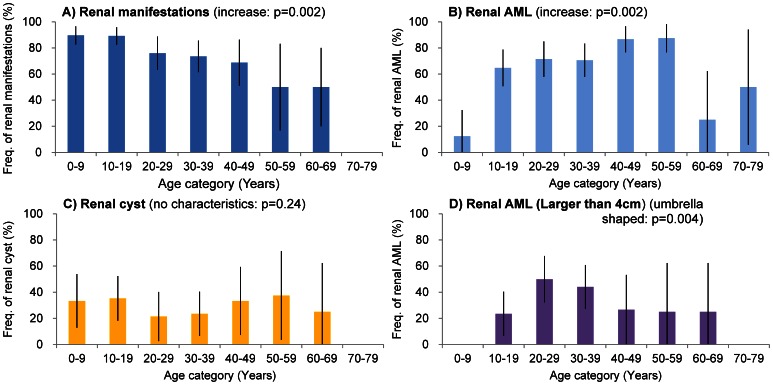
Frequency of renal manifestations, different-sized renal angiomyolipomas and cysts in each age group. The frequency (%) of patients with A) renal manifestations (blue), B) renal AML (angiomyolipomas: light blue), C) renal cysts (yellow) and D) renal AML larger than 4 cm (purple), as stratified by age. Renal manifestations include any renal symptom. Renal AML includes any sized renal AML. The frequency (%) of patients with each manifestation in each examined age group. The bars indicate the CI.

The renal manifestations of TSC include renal angiomyolipomas (AML), renal cysts and, rarely, carcinomas. The rate of renal lesions observed in our patients overall (71%) was similar to the previously reported prevalence (Table 1) [6], [7], [8], [9], [34], [35], [36]. The frequency of renal AML (61%), cysts (28%) and renal-cell carcinoma (2.6%) among our subjects were also similar to those observed in previous reports [6], [7], [8], [9], [36] (Table 1). Considering the progression of the frequency of renal AML and cysts, renal AML might grow rapidly during the teen years of age as reported previously [37], although the frequency of renal cysts did not change during the patients’ lifetimes (Fig. 4B, C). Among the 92 AML-positive patients, 44 (48%) had AMLs larger than 4 cm in diameter. In contrast, among the patients younger than 9 years of age, all AMLs were smaller than 4 cm. The frequency of AMLs larger than 4 cm was 24% for teenaged patients and 50% for patients in their twenties. The frequency of renal AMLs larger than 4 cm peaked in patients aged 20–29 years of age and then decreased. This association was also significant (Fig. 4 D). One reason for this result was that the TSC patients with slight renal manifestations over 30 years of age were newly diagnosed. Another reason might be due to treatment. Because being larger than 4 cm and rich in blood vessels or aneurysms increases the risk of rupture in AMLs [38], AMLs larger than 4 cm were treated in our hospital to prevent rupture (Fig. 4B, D). The complications of both AML and cysts occurred in 18% of patients. In the 24 patients younger than 9 years of age, 63% had no renal manifestations (*p*<0.0001), 33% had cysts, 12% had AML, and only 8% had both AML and cysts. In contrast, of the 34 teenaged patients, 65% had AML (*p*<0.0001), 36% had renal cysts, and 21% had no manifestations. Complications of both AML and cysts tended to increase in the second decade; however, there was no significant association between age and complications of AML and cysts.

### Pulmonary Manifestations

**Figure 5:**
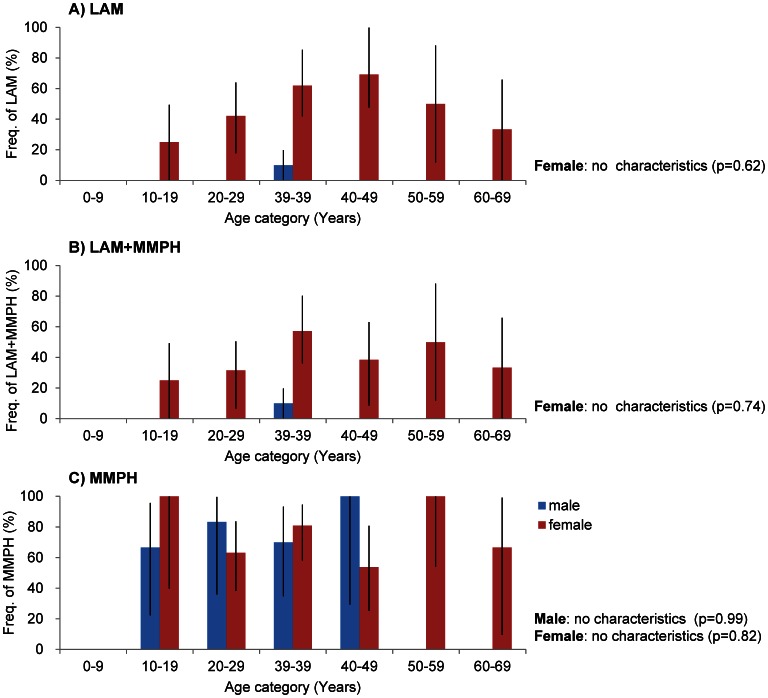
Frequency of each pulmonary manifestation stratified by age and gender. LAM and MMPH stand for lymphangioleiomyomatosis and multifocal micronodular pneumocyte hyperplasia, respectively. A) The frequency of patients with LAM with/without MMPH, B) with both LAM and MMPH and C) with MMPH with/without LAM by age and gender are shown. The blue column indicates males, and the red column indicates females. The frequency (%) of patients with each manifestation in each examined age group. The bars indicate the CI.

LAM and MMPH are the pulmonary manifestations of TSC. Among the 95 patients examined, 75 patients (79%) experienced pulmonary manifestations, with 37 (39%) having LAM, 67 (71%) having MMPH and 29 (31%) having both LAM and MMPH (Table 1). The reported prevalence of LAM varies from approximately 2% in early reports [39], [40], [41] to 40% in several recent reports [14], [42], [43]. The frequency of LAM in our patients who had been diagnosed with HRCT was 39%, which is similar to recent studies (Table 1). Among the 67 female patients, 36 (54%) had LAM, 48 (72%) had MMPH, and 28 (42%) had both. Among the 28 male patients, 1 (3.6%) had LAM complicated with MMPH, 19 (67.9%) had MMPH, and the remaining 9 (32%) did not have any pulmonary manifestation (Fig. 5). The frequency of LAM in females was significantly higher than in males (*p* = 0.0015). The rate of LAM tended to increase after 20 years of age and was highest in the group in their forties; however, contrary to many previous reports, the prevalence of LAM had no association with age (Fig. 5A). MMPH, which is another pulmonary manifestation of TSC, had no association with age and gender (Fig. 5C). Because 78% of the LAM patients had complications with MMPH and only 8 of the 37 LAM patients (21%) had LAM alone, the ratio of TSC-LAM with MMPH is significantly higher than that of TSC-LAM without MMPH (p<0.001). As we could not find any reports about sporadic-LAM with MMPH, the complications with MMPH might be characteristic of TSC-LAM and the difference between sporadic-LAM. Of the 37 LAM patients who were diagnosed with HRCT and precise lung function tests, only 7 patients (18.8%) became severe during a subsequent 10-year observation period, and the remaining 30 patients remained in stable condition (data not shown).

### Skin Manifestations

Skin lesions are the most frequently observed manifestations of TSC [14], [44], [45], [46], [47] The skin manifestations of TSC include AFs, forehead plaques, hypomelanotic macules, shagreen patches and ungual fibromas. In our study, among the 166 patients studied, 99% of the patients had some type of skin symptom (Table 1). Only 2 patients (1.2%), a 3-year-old female and a 38-year-old male, had no skin lesions. Both patients had familial TSC, and the male patient, who had a son with typical TSC, was diagnosed by genetic analysis.

**Figure 6:**
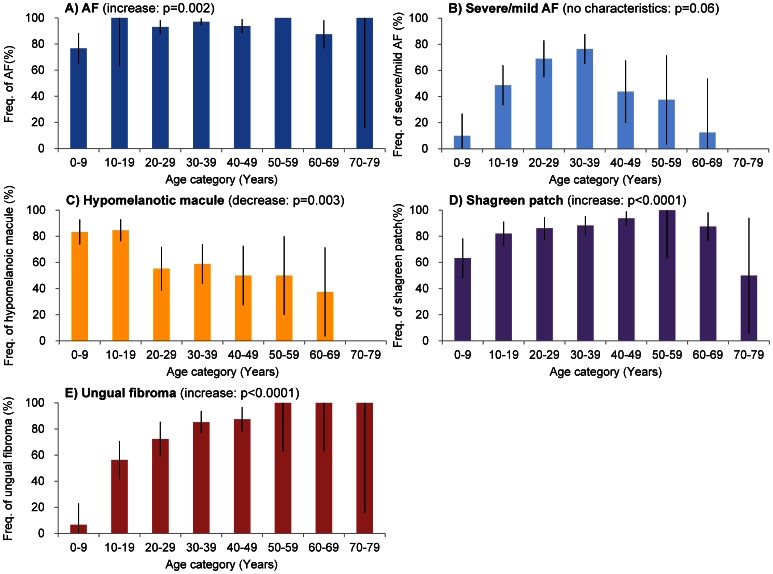
Trends in the age-specific frequency of each skin manifestation. The frequency indicates the proportion of patients in each age group with A) FA (facial angiofibromas: blue), B) severe and mild FA (light blue), C) hypomelanotic macules (yellow), D) shagreen patches (purple) and E) ungual fibromas (red). The angiofibromas were divided into three classes, slight, mild and severe, as described in the text. AF includes angiofibromas of all classes. The bars indicate the CI.

AF is the most emblematic symptom of TSC because of its prominent location on the face. Among our patients, 93% were affected by AFs. This frequency was significantly higher than in previous reports [7], [14], [45], [46], [48], [49] (Table 1). AFs appeared in patients younger than 9 years as small papules or telangiectasis. Although the frequency of AF among the patients younger than 9 years was slightly low (77%), the frequency of AF was greater than 87% for all the other age groups (Fig. 6A). The frequency of severe to mild AF increased as patients aged; it was the highest in the fourth decade and decreased after the fifth decade (Fig. 6B).

The reported prevalence of forehead plaques varies from 12 to 40% [7], [8], [9], [12], [14], [34], [45], [46], [49], [50]. The frequency of forehead plaques was 46% in our patients, which is a little higher than in previous reports; however, the difference was not significant (Table 1). All patients with a forehead plaque had AFs. Usually, forehead plaques appeared at birth as flat, purplish brown-red macules that rose gradually to form plaques. Well-vascularized plaques are sometimes difficult to distinguish from severe AFs. The plaques occasionally appeared at sites other than the forehead.

Another common skin lesion in TSC patients is hypomelanotic macules. Hypomelanotic macules are thought to be present in more than 90% of TSC patients [44], [46], [47], [48], [51]. Only 65% of our patients had three or more hypomelanotic macules, which was a significantly lower frequency than previously reported (Table 1). The frequency of three or more hypomelanotic macules among patients younger than 19 years was greater than 80% and decreased with age (Fig. 6C). This trend was significant. Hypomelanotic macules appeared in the younger subjects and faded or disappeared as these patients became older (Fig. 6C). A total of 109 patients (66%) had three or more hypomelanotic macules, 17 (10%) had two hypomelanotic macules, 11 (6%) had one hypomelanotic macule, and 31 (18%) had no hypomelanotic macules. According to previous reports that examined 423 healthy individuals, the prevalence of one hypomelanotic macule is 16 (3.8%), that of two hypomelanotic macules is 3 (0.7%) and that of three or more hypomelanotic macules is 1 (0.24%) in the general population; no healthy individuals with more than four hypomelanotic macules have been reported [52]. Considering these results, the frequency of hypomelanotic macules was higher among TSC patients than in the healthy population, and the frequency of three or more hypomelanotic macules was significantly higher than in the healthy population (*p*<0.0001) and was characteristic of TSC.

The frequency of shagreen patches in our study (83%) was higher than that in previous reports [14], [44], [45], [46], [47], [48] (Table 1). Shagreen patches were observed in 63% of the patients younger than 9 years of age, and the frequency increased with age, and the increasing tendency was significant (Fig. 6D). Shagreen patches exhibit various atypical appearances. Some patients are misdiagnosed with verrucas (warts). Other atypical shagreen patches appear similar to huge collagen hamartomas as reported by Torrelo et al. [53]. Among the 166 patients in our study population, 8 had huge protuberant collagen hamartomas (4.8%). One of these patients had been misdiagnosed as having a desmoid. The prevalence of shagreen patches may have been underestimated due to misdiagnosis. Misdiagnosis may also be one reason for the low prevalence of shagreen patches reported in the literature.

The reported prevalence of ungual fibromas in TSC patients varies from 15 to 80% [14], [45], [46], [48], [54]. Most of this variability appears to be attributable to differences in the age compositions of the study populations. In the present study, the frequency of ungual fibromas was 64% and was similar to that in previous reports (Table 1). In this work, ungual fibromas appeared during the teenage years. The frequency increased with age, reaching approximately 85% in the fourth decade and 100% in the sixth decade (Fig. 6E). In the 106 patients with ungual fibroma, the rate of having three or more ungual fibromas, two ungual fibromas, and one ungual fibroma were 76% (80), 13% (14) and 11% (12), respectively. There were no patients younger than 10 years of age with more than one ungual fibroma. Seventy-six percent of the patients with ungual fibromas had more than three, and this proportion was significant (*p*<0.001). Although ungual fibroma is important for the diagnosis of TSC [55], as acquired ungual fibromas are uncommon [56], [57], the presence of more than three ungual fibromas is a stronger indicator of a TSC diagnosis.

### Cardiac Manifestations

**Figure 7:**
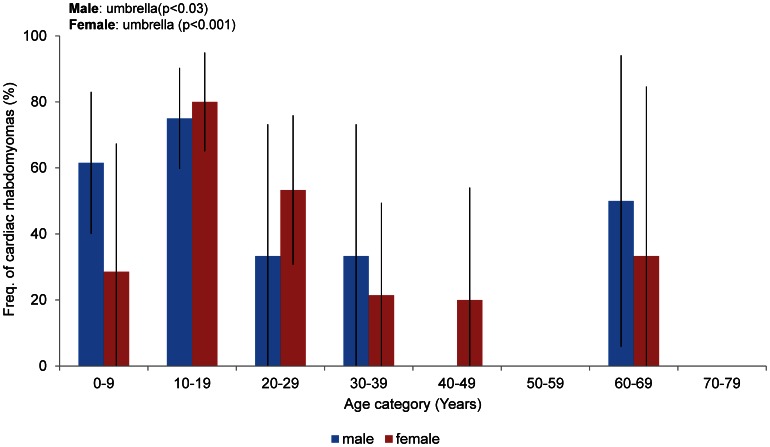
Frequency of cardiac rhabdomyomas stratified by age and gender. The rates indicate the percentages of female patients with cardiac rhabdomyomas (red) and male patients with cardiac rhabdomyomas (blue). The bars indicate the CI.

Echocardiography was used to examine 106 patients (45 males and 61 females), including all patients with histories of cardiac rhabdomyoma. Fifty-four patients (51%) had no current symptoms. Forty-nine patients (46%) had ongoing rhabdomyoma (Table 1). Only 3 patients (2.8%) had cardiac manifestations without cardiac rhabdomyoma; 2 of these had electrocardiographic abnormalities (WPW syndrome and atrial extrasystoles). The third patient had a ventricular septal defect (VSD). In our study, as in previous reports [58], 46% of the TSC patients had cardiac rhabdomyomas (Table 1), and almost all had no current symptoms. The prevalence of cardiac rhabdomyoma was 50% in the patients younger than 10 years of age. The prevalence peaked in the teens (77%) and then decreased. This transition was significant (Fig. 7). The frequency of cardiac rhabdomyoma decreased in women older than 30 years of age compared with the groups younger than 29 years of age (*p* = 0.006). Cardiac rhabdomyomas have been reported in 40–60% of TSC patients [7], [8], [9], [12], [14] and are more common among patients younger than 2 years of age [58]. This tumor develops prenatally, can be visualized at 22–28 weeks of gestation, is clinically silent in many cases and tends to regress [59]; however, cardiac rhabdomyomas may appear *de novo* or may grow in some cases [58]. The relatively large number of female patients who had cardiac rhabdomyomas in their twenties in our study indicates the growth or *de novo* appearance of cardiac rhabdomyomas in this group, as described above.

### Uterine and Ovarian Manifestations

**Figure 8:**
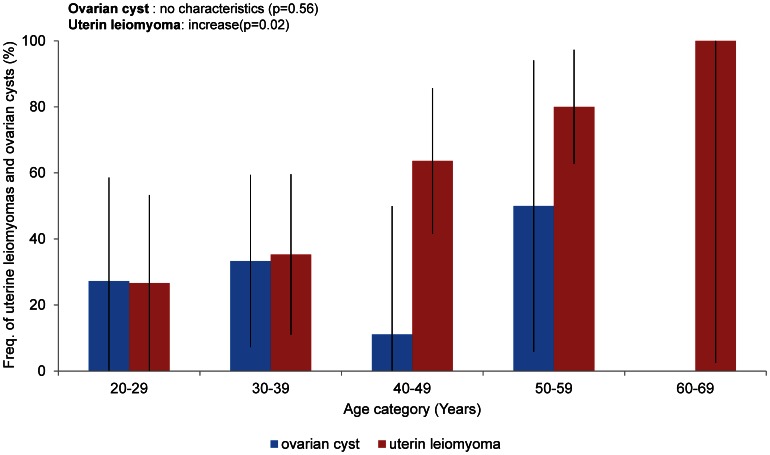
Frequency of uterine leiomyomas and ovarian cysts in each age group. Fifty-one and 39 female patients over 20 years of age were examined for uterine leiomyomas and ovarian cysts, respectively. The rates indicate the proportions of patients in each age group with uterine leiomyomas (red) and ovarian cysts (blue). The bars indicate the CI.

Examinations of 51 female patients over 20 years of age revealed that 29 patients (57%) displayed uterine manifestations of TSC (Table 1). Of these patients, 24 (47%) had a uterine leiomyoma, 2 had cysts, and the remaining 3 had carcinoma, PEComa and uterine bleeding of unknown cause, respectively. A previous study reported that Eker rats (TSC2^+/EK^) with a mutation in *TSC2* developed uterine leiomyomas [60]. Recently, the increased expression of tuberin in human uterine leiomyomas has been reported [61]. We investigated the incidence of uterine leiomyomas among TSC patients. The rate of uterine leiomyomas in patients older than 20 years was 47%. The frequency of uterine leiomyomas increased in patients older than 40 years of age (Fig. 8). Human uterine leiomyoma is a benign smooth-muscle tumor, and the incidence of this tumor in the general population reaches 25–45% in females of reproductive age [62], [63]. The frequency of uterine leiomyomas in our TSC subjects tended to be greater than that in general population (*p* = 0.1493). This difference was not significant, but the trend may suggest a need for further study.

Recently, relationships among TSC, LAM and PEComa have been reported, including a uterine PEComa in a TSC patient [64]. We also observed a case of uterine PEComa that had been initially diagnosed as uterine leiomyoma with abdominal MRI and was then diagnosed as uterine PEComa by histological examination. It is difficult to differentiate uterine PEComa from uterine leiomyoma using MRI without a histopathological examination. Among the 24 patients who were diagnosed with uterine leiomyomas using abdominal MRI, 3 patients with unusually huge multilocular tumors were considered to have PEComa. It may be important to investigate a possible association between uterine PEComa and TSC.

Examinations of 39 female patients over 20 years of age revealed that the frequency of ovarian cysts in this group was 28% (Table 1) and had no relationship with age (Fig. 8).

### Endocrine Manifestations

Recently, a relationship between endocrine tumors and TSC has also been reported [65]. Of 95 examined individuals (28 males and 67 females), 26 (27%) had an observable abnormality of the thyroid gland. The numbers of thyroid-gland abnormalities among male and female patients were 5 (18%) and 21 (31%), respectively. Of the 26 patients with thyroid defects, 4 had functional abnormalities, and the remainder had normal thyroid function. Only 2 patients had cysts. Eighteen patients had adenomas of the thyroid gland, and all patients who underwent needle biopsies were diagnosed with an adenomatous goiter, although papillary adenomas are known to be the most common thyroid lesions in TSC patients [66].

Regarding other endocrine neoplasms, such as parathyroid tumors, pancreatic tumors, pituitary tumors or adrenal tumors, 9 of the 95 examined individuals (10%) had pancreatic manifestations, one patient had an adrenal tumor and the remaining patient had a parathyroid tumor. Approximately nine patients had pancreatic manifestations. Four patients had pancreatic tumors, and two patients had dilatation of the main pancreatic duct. One patient had calcifications, and one patient had cyst and pancreatic enlargement each. In addition, six patients had mammary tumors. No patients had more than two endocrine neoplasms. This result is useful for differentiating TSC and multiple endocrine neoplasm type 1 (MEN1) [65], which is associated with AF and collagenoma and more than 2 endocrine tumors.

### Genetic Analysis

Genetic analyses were performed for 75 patients (67 families), and mutations were identified in 45 patients (60%). Mutations in *TSC1* were found in 21 patients (28%), and mutations in *TSC2* were found in 24 patients (32%). In the 21 patients with *TSC1* mutations, 3 familial cases (7 individuals) were included, and in the 24 patients with *TSC2* mutations, 2 familial cases (5 individuals) were included. No genetic mutations were identified in the remaining 30 patients, including in 3 of the familial cases (5 individuals). Several researchers have searched for mutations in the *TSC1* and *TSC2* genes among TSC patients. Mutations in these genes have been detected in approximately 80% of TSC patients [5], [7], [8], [9]. In our study, the rate (40%) of patients with no identifiable mutations was significantly higher than the rate obtained in previous reports (*p*<0.001). Although many reports have suggested that the prevalence of *TSC2* mutations is higher than that of *TSC1* mutations [7], [8], [9], we observed that these rates were approximately equal (*p* = 0.59). There was no significant difference between the prevalences of three genotypes of *TSC1, TSC2* and the “no mutation identified” (*NMI*; *p* = 0.35) genotype in our data. The rates of intellectual disability in the TSC1, TSC2 and NMI groups were 26%, 46% and 63%, respectively. There was no significant association between intellectual disability and the *TSC1* and *TSC2* genotypes (*p* = 0.16, *p* = 0.77); however, the *NMI* genotype was significantly associated with intellectual disability (*p* = 0.03). In particular, the rate of severe intellectual disability (level 3) was 13% in the NMI group, and no patients with severe intellectual disabilities were found in the TSC1 and TSC2 groups. The previously reported prevalences of the *TSC1, TSC2* and *NMI* genotypes were 28%, 32%, and 15% [9]; 12.5%, 87%, and 17% [7]; 17%, 50%, and 33% [8] and 15%, 66%, and 20% [5], respectively. Compared with previous data, there was no significant difference in the frequency of *TSC1* (Table1). The frequency of *TSC2* was significantly low (Table1), and the frequency of *NMI* was significantly high (Table1). The previously reported prevalences of intellectual disability in each genotype *TSC1, TSC2, NMI* were 49%, 83%, 36% [9]; 50%, 71%, 54% [7] and 45%, 57%, 39% [8], respectively. Compared with the previous data, the frequency of intellectual disability was significantly lower for the *TSC1* and *TSC2* genotypes (*p* = 0.009, *p* = 0.02) and significantly higher for the *NMI* genotype (*p* = 0.02).

NMI patients have also been reported to have milder neurological features of TSC [7], [9]; however, in our study, the NMI cases had more severe intellectual disabilities compared with the patients with *TSC1* or *TSC2* mutations.

### Conclusions

We examined the current frequency and characteristics of TSC manifestations in a Japanese population that included adults and children to enhance our understanding of the current manifestations and natural history of TSC; these results will be used to support the appropriate management of TSC patients. We statistically analyzed the current frequency of TSC manifestations compared with historical data (Table 1). We also analyzed the association of the frequency of manifestation with age (Figs. 1, 2, 4, 5, 6, 7, 8). The frequencies of hypomelanotic macules and most of the neurological symptoms of TSC (such as SEGA, epilepsy, refractory epilepsy, intellectual disability) were lower in this study than in other studies. These manifestations are present at high frequency in younger patients and become less common with age (Figs. 2, 6). In contrast, the frequency of TSC manifestations that tend to increase with age, such as AF and shagreen patches, were greater than in other studies. These results indicated that the proportion of the patients who were overlooked in childhood and newly diagnosed in adulthood had increased. For those patients, careful attention must be paid to renal, pulmonary and skin manifestations as proxies for neurological manifestations. Another possibility is that the differences observed are related to some patients seeking dermatological treatment for TSC skin lesions and some patients seeking neurological symptoms such as epilepsy, mental retardation and autism.

Another important manifestation is the rare disease LAM, which is characterized by the proliferation of abnormal muscle-like cells (LAM cells). There is currently no effective treatment for LAM. The clinical signs and symptoms are dyspnea, recurrent pneumothorax, hemoptysis and ultimately respiratory failure; however, no symptoms appear during the early stages. HRCT or precise lung-function tests are the only methods to evaluate early LAM. LAM is a relatively novel disease compared with the other TSC manifestations. Although the frequency of LAM in this study was high, it was the same as the frequency observed in a previous report (Table 1). In this study, almost 40% of all examined patients and 54% of female patients were suffering from LAM. Although the frequency of LAM was high in patients with TSC, the conditions of 19% of the LAM patients worsened rapidly. The remaining 81% of the LAM patients did not progress or worsened slowly and thus did not require treatment. Although the proportion of patients with severe LAM might be smaller than that of patients with a mild disease, there is no way to distinguish severe LAM from mild LAM in the early stages of the disease. Therefore, follow-up of all patients with LAM is necessary. Although the prevalence of LAM was thought to be high in women in their 30 s and older, in this study, the frequency of LAM displayed no significant association with age (Fig. 5). In addition, the youngest LAM patient among our cases was a 13-year-old girl, and there was also one male LAM patient. Thus, careful attention must be paid to both younger patients and male patients. Unlike sporadic LAM, TSC-LAM may be associated with MMPH.

Another finding in this study was the importance of skin disease. Ninety-nine percent of TSC patients had some type of skin disease. As mentioned above, the frequency of hypomelanotic macules was higher among TSC patients than in the healthy population, and three or more hypomelanotic macules were a significant characteristic of TSC. Although hypomelanotic macules are not specific to TSC, they are considered an important symptom in the early diagnosis of TSC because of their higher frequency and early appearance in TSC patients. The frequency of AF was high and displayed no significant association with age (Fig. 6 1). Considering these results, AF appeared earlier than predicted, but the picture may be mild. Ungual fibroma was a characteristic skin manifestation that appeared later than other skin manifestations and increased in frequency with age (Fig. 6 E). More than three ungual fibromas were significant indicators of TSC. Considering the above results, the importance of skin manifestations as diagnostic signs was reconfirmed. In addition, our data indicate a high frequency of uterine leiomyoma among TSC patients and a need to pay attention to the possibility of uterine PEComa.

This is the first epidemiological study of Japanese TSC patients to include a broad sampling of the population and various manifestations. The frequency of the manifestations revealed in this work reflects current trends among TSC patients in Japan.

## References

[1] Osborne JP, Jones AC, Burley MW, Jeganathan D, Young J, et al (2000) Non-penetrance in tuberous sclerosis. Lancet 355: 1698. 10905251 10.1016/s0140-6736(00)02247-9

[2] van Slegtenhorst M, de Hoogt R, Hermans C, Nellist M, Janssen B, et al (1997) Identification of the tuberous sclerosis gene TSC1 on chromosome 9q34. Science 277: 805–808. 9242607 10.1126/science.277.5327.805

[3] Identification and characterization of the tuberous sclerosis gene on chromosome 16. Cell 75: 1305–1315. 10.1016/0092-8674(93)90618-z 8269512

[4] Chu-Shore CJ, Major P, Camposano S, Muzykewicz D, Thiele EA (2010) The natural history of epilepsy in tuberous sclerosis complex. Epilepsia 51: 1236–1241. 20041940 10.1111/j.1528-1167.2009.02474.x PMC3065368

[5] Jones AC, Shyamsundar MM, Thomas MW, Maynard J, Idziaszczyk S, et al (1999) Comprehensive mutation analysis of TSC1 and TSC2-and phenotypic correlations in 150 families with tuberous sclerosis. Am J Hum Genet 64: 1305–1315. 10205261 10.1086/302381 PMC1377866

[6] Rakowski SK, Winterkorn EB, Paul E, Steele DJ, Halpern EF, et al (2006) Renal manifestations of tuberous sclerosis complex: Incidence, prognosis, and predictive factors. Kidney Int 70: 1777–1782. 17003820 10.1038/sj.ki.5001853

[7] Dabora SL, Jozwiak S, Franz DN, Roberts PS, Nieto A, et al (2001) Mutational analysis in a cohort of 224 tuberous sclerosis patients indicates increased severity of TSC2, compared with TSC1, disease in multiple organs. Am J Hum Genet 68: 64–80. 11112665 10.1086/316951 PMC1234935

[8] Au KS, Williams AT, Roach ES, Batchelor L, Sparagana SP, et al (2007) Genotype/phenotype correlation in 325 individuals referred for a diagnosis of tuberous sclerosis complex in the United States. Genet Med 9: 88–100. 17304050 10.1097/gim.0b013e31803068c7

[9] Sancak O, Nellist M, Goedbloed M, Elfferich P, Wouters C, et al (2005) Mutational analysis of the TSC1 and TSC2 genes in a diagnostic setting: genotype–phenotype correlations and comparison of diagnostic DNA techniques in Tuberous Sclerosis Complex. Eur J Hum Genet 13: 731–741. 15798777 10.1038/sj.ejhg.5201402

[10] Chopra M, Lawson JA, Wilson M, Kennedy SE, Taylor P, et al (2011) An Australian tuberous sclerosis cohort: are surveillance guidelines being met? J Paediatr Child Health 47: 711–716. 21449900 10.1111/j.1440-1754.2011.02038.x

[11] Hong CH, Darling TN, Lee CH (2009) Prevalence of tuberous sclerosis complex in Taiwan: a national population-based study. Neuroepidemiology 33: 335–341. 19887839 10.1159/000254569

[12] Yates JR, Maclean C, Higgins JN, Humphrey A, le Marechal K, et al.. (2011) The Tuberous Sclerosis 2000 Study: presentation, initial assessments and implications for diagnosis and management. Arch Dis Child. 10.1136/adc.2011.211995 21813552

[13] Staley BA, Vail EA, Thiele EA (2011) Tuberous sclerosis complex: diagnostic challenges, presenting symptoms, and commonly missed signs. Pediatrics 127: e117–125. 21173003 10.1542/peds.2010-0192 PMC3010088

[14] Hallett L, Foster T, Liu Z, Blieden M, Valentim J (2011) Burden of disease and unmet needs in tuberous sclerosis complex with neurological manifestations: systematic review. Curr Med Res Opin 27: 1571–1583. 21692602 10.1185/03007995.2011.586687

[15] Roach ES, Gomez MR, Northrup H (1998) Tuberous sclerosis complex consensus conference: revised clinical diagnostic criteria. J Child Neurol 13: 624–628. 9881533 10.1177/088307389801301206

[16] Sasongko TH, Wataya-Kaneda M, Koterazawa K, Gunadi, Yusoff S, et al (2008) Novel mutations in 21 patients with tuberous sclerosis complex and variation of tandem splice-acceptor sites in TSC1 exon 14. Kobe J Med Sci 54: E73–81. 18772611

[17] Curatolo P, Bombardieri R, Jozwiak S (2008) Tuberous sclerosis. Lancet 372: 657–668. 18722871 10.1016/S0140-6736(08)61279-9

[18] Thiele EA (2004) Managing epilepsy in tuberous sclerosis complex. J Child Neurol 19: 680–686. 15563014 10.1177/08830738040190090801

[19] Jozwiak S, Schwartz RA, Janniger CK, Bielicka-Cymerman J (2000) Usefulness of diagnostic criteria of tuberous sclerosis complex in pediatric patients. J Child Neurol 15: 652–659. 11063078 10.1177/088307380001501003

[20] Devlin LA, Shepherd CH, Crawford H, Morrison PJ (2006) Tuberous sclerosis complex: clinical features, diagnosis, and prevalence within Northern Ireland. Dev Med Child Neurol 48: 495–499. 16700943 10.1017/S0012162206001058

[21] Holmes GL, Stafstrom CE (2007) Tuberous sclerosis complex and epilepsy: recent developments and future challenges. Epilepsia 48: 617–630. 17386056 10.1111/j.1528-1167.2007.01035.x

[22] Franz DN, Bissler JJ, McCormack FX (2010) Tuberous sclerosis complex: neurological, renal and pulmonary manifestations. Neuropediatrics 41: 199–208. 21210335 10.1055/s-0030-1269906 PMC4629839

[23] Cass H, Sekaran D, Baird G (2006) Medical investigation of children with autistic spectrum disorders. Child Care Health Dev 32: 521–533. 16919131 10.1111/j.1365-2214.2006.00630.x

[24] de Vries PJ, Hunt A, Bolton PF (2007) The psychopathologies of children and adolescents with tuberous sclerosis complex (TSC): a postal survey of UK families. Eur Child Adolesc Psychiatry 16: 16–24. 17268883 10.1007/s00787-006-0570-3

[25] Hunt A, Shepherd C (1993) A prevalence study of autism in tuberous sclerosis. J Autism Dev Disord 23: 323–339. 8331050 10.1007/BF01046223

[26] Smalley SL, Tanguay PE, Smith M, Gutierrez G (1992) Autism and tuberous sclerosis. J Autism Dev Disord 22: 339–355. 1400103 10.1007/BF01048239

[27] Webb DW, Fryer AE, Osborne JP (1991) On the incidence of fits and mental retardation in tuberous sclerosis. J Med Genet 28: 395–397. 1870096 10.1136/jmg.28.6.395 PMC1016904

[28] Jozwiak S, Kotulska K, Domanska-Pakiela D, Lojszczyk B, Syczewska M, et al (2011) Antiepileptic treatment before the onset of seizures reduces epilepsy severity and risk of mental retardation in infants with tuberous sclerosis complex. Eur J Paediatr Neurol 15: 424–431. 21507691 10.1016/j.ejpn.2011.03.010

[29] Jozwiak S, Goodman M, Lamm SH (1998) Poor mental development in patients with tuberous sclerosis complex: clinical risk factors. Arch Neurol 55: 379–384. 9520012 10.1001/archneur.55.3.379

[30] Shepherd CW, Stephenson JB (1992) Seizures and intellectual disability associated with tuberous sclerosis complex in the west of Scotland. Dev Med Child Neurol 34: 766–774. 1526347 10.1111/j.1469-8749.1992.tb11515.x

[31] Chou PC, Chang YJ (2004) Prognostic factors for mental retardation in patients with tuberous sclerosis complex. Acta Neurol Taiwan 13: 10–13. 15315295

[32] Joinson C, O'Callaghan FJ, Osborne JP, Martyn C, Harris T, et al (2003) Learning disability and epilepsy in an epidemiological sample of individuals with tuberous sclerosis complex. Psychol Med 33: 335–344. 12622312 10.1017/s0033291702007092

[33] Jansen FE, Vincken KL, Algra A, Anbeek P, Braams O, et al (2008) Cognitive impairment in tuberous sclerosis complex is a multifactorial condition. Neurology 70: 916–923. 18032744 10.1212/01.wnl.0000280579.04974.c0

[34] Baskin HJ Jr (2008) The pathogenesis and imaging of the tuberous sclerosis complex. Pediatr Radiol 38: 936–952. 18414839 10.1007/s00247-008-0832-y

[35] Castagnetti M, Vezzu B, Laverda A, Zampieri S, Rigamonti W (2007) Urological counseling and followup in pediatric tuberous sclerosis complex. J Urol 178: 2155–2159. 17870119 10.1016/j.juro.2007.07.058

[36] Bernstein J, Robbins TO (1991) Renal involvement in tuberous sclerosis. Ann N Y Acad Sci 615: 36–49. 2039157 10.1111/j.1749-6632.1991.tb37746.x

[37] Ewalt DH, Sheffield E, Sparagana SP, Delgado MR, Roach ES (1998) Renal lesion growth in children with tuberous sclerosis complex. J Urol 160: 141–145. 9628635

[38] Yamakado K, Tanaka N, Nakagawa T, Kobayashi S, Yanagawa M, et al (2002) Renal angiomyolipoma: relationships between tumor size, aneurysm formation, and rupture. Radiology 225: 78–82. 12354988 10.1148/radiol.2251011477

[39] Hancock E, Osborne J (2002) Lymphangioleiomyomatosis: a review of the literature. Respir Med 96: 1–6. 11863203 10.1053/rmed.2001.1207

[40] Castro M, Shepherd CW, Gomez MR, Lie JT, Ryu JH (1995) Pulmonary tuberous sclerosis. Chest 107: 189–195. 7813275 10.1378/chest.107.1.189

[41] Osborne JP, Fryer A, Webb D (1991) Epidemiology of tuberous sclerosis. Ann N Y Acad Sci 615: 125–127. 2039137 10.1111/j.1749-6632.1991.tb37754.x

[42] Adriaensen ME, Schaefer-Prokop CM, Duyndam DA, Zonnenberg BA, Prokop M (2011) Radiological evidence of lymphangioleiomyomatosis in female and male patients with tuberous sclerosis complex. Clin Radiol 66: 625–628. 21459371 10.1016/j.crad.2011.02.009

[43] Urban T, Lazor R, Lacronique J, Murris M, Labrune S, et al (1999) Pulmonary lymphangioleiomyomatosis. A study of 69 patients. Groupe d'Etudes et de Recherche sur les Maladies “Orphelines” Pulmonaires (GERM”O”P). Medicine (Baltimore) 78: 321–337. 10499073 10.1097/00005792-199909000-00004

[44] Sogut A, Ozmen M, Sencer S, Caliskan M, Aydinli N, et al (2002) Clinical features of tuberous sclerosis cases. Turk J Pediatr 44: 98–101. 12026215

[45] Jozwiak J, Galus R (2008) Molecular implications of skin lesions in tuberous sclerosis. Am J Dermatopathol 30: 256–261. 18496427 10.1097/DAD.0b013e31816e22a5

[46] Jozwiak S, Schwartz RA, Janniger CK, Michalowicz R, Chmielik J (1998) Skin lesions in children with tuberous sclerosis complex: their prevalence, natural course, and diagnostic significance. Int J Dermatol 37: 911–917. 9888331 10.1046/j.1365-4362.1998.00495.x

[47] Hake S (2011) Cutaneous manifestations of tuberous sclerosis. Ochsner J 10: 200–204. PMC3096212 21603378

[48] Webb DW, Clarke A, Fryer A, Osborne JP (1996) The cutaneous features of tuberous sclerosis: a population study. Br J Dermatol 135: 1–5. 8776349

[49] Schwartz RA, Fernandez G, Kotulska K, Jozwiak S (2007) Tuberous sclerosis complex: advances in diagnosis, genetics, and management. J Am Acad Dermatol 57: 189–202. 17637444 10.1016/j.jaad.2007.05.004

[50] Sun XF, Yan CL, Fang L, Shen FM, Liao KH (2005) Cutaneous lesions and visceral involvement of tuberous sclerosis. Chin Med J (Engl) 118: 215–219. 15740650

[51] Juvet SC, McCormack FX, Kwiatkowski DJ, Downey GP (2007) Molecular pathogenesis of lymphangioleiomyomatosis: lessons learned from orphans. Am J Respir Cell Mol Biol 36: 398–408. 17099139 10.1165/rcmb.2006-0372TR PMC2176113

[52] Vanderhooft SL, Francis JS, Pagon RA, Smith LT, Sybert VP (1996) Prevalence of hypopigmented macules in a healthy population. J Pediatr 129: 355–361. 8804323 10.1016/s0022-3476(96)70066-5

[53] Torrelo A, Hadj-Rabia S, Colmenero I, Piston R, Sybert VP, et al (2012) Folliculocystic and collagen hamartoma of tuberous sclerosis complex. J Am Acad Dermatol 66: 617–621. 21839539 10.1016/j.jaad.2011.04.002

[54] Aldrich CS, Hong CH, Groves L, Olsen C, Moss J, et al (2009) Acral lesions in tuberous sclerosis complex: insights into pathogenesis. J Am Acad Dermatol 63: 244–251. 10.1016/j.jaad.2009.08.042 PMC2947366 20462663

[55] Zeller J, Friedmann D, Clerici T, Revuz J (1995) The significance of a single periungual fibroma: report of seven cases. Arch Dermatol 131: 1465–1466. 10.1001/archderm.131.12.1465b 7492148

[56] Carlson RM, Lloyd KM, Campbell TE (2007) Acquired periungual fibrokeratoma: a case report. Cutis 80: 137–140. 17944173

[57] Cahn RL (1977) Acquired periungual fibrokeratoma. A rare benign tumor previously described as the garlic-clove fibroma. Arch Dermatol 113: 1564–1568. 931397 10.1001/archderm.113.11.1564

[58] Jozwiak S, Kotulska K, Kasprzyk-Obara J, Domanska-Pakiela D, Tomyn-Drabik M, et al (2006) Clinical and genotype studies of cardiac tumors in 154 patients with tuberous sclerosis complex. Pediatrics 118: e1146–1151. 16940165 10.1542/peds.2006-0504

[59] Kotulska K, Larysz-Brysz M, Grajkowska W, Jozwiak J, Wlodarski P, et al (2009) Cardiac rhabdomyomas in tuberous sclerosis complex show apoptosis regulation and mTOR pathway abnormalities. Pediatr Dev Pathol 12: 89–95. 17990907 10.2350/06-11-0191.1

[60] Walker CL, Hunter D, Everitt JI (2003) Uterine leiomyoma in the Eker rat: a unique model for important diseases of women. Genes Chromosomes Cancer 38: 349–356. 14566855 10.1002/gcc.10281

[61] Cui L, Ren Y, Yin H, Wang Y, Li D, et al (2011) Increased expression of tuberin in human uterine leiomyoma. Fertil Steril 95: 1805–1808. 21145542 10.1016/j.fertnstert.2010.11.028

[62] Luciano AA (2009) Myomectomy. Clin Obstet Gynecol 52: 362–371. 19661752 10.1097/GRF.0b013e3181b0bdcd

[63] Baird DD, Dunson DB, Hill MC, Cousins D, Schectman JM (2003) High cumulative incidence of uterine leiomyoma in black and white women: ultrasound evidence. Am J Obstet Gynecol 188: 100–107. 12548202 10.1067/mob.2003.99

[64] Lim GS, Oliva E (2011) The morphologic spectrum of uterine PEC-cell associated tumors in a patient with tuberous sclerosis. Int J Gynecol Pathol 30: 121–128. 21293289 10.1097/PGP.0b013e3181fa5a99

[65] Piecha G, Chudek J, Wiecek A (2008) Multiple Endocrine Neoplasia type 1. Eur J Intern Med 19: 99–103. 18249304 10.1016/j.ejim.2007.08.004

[66] Perou ML, Gray PT (1960) Mesenchymal hamartomas of the kidney. J Urol 83: 240–261. 14431942 10.1016/S0022-5347(17)65698-2

