# Correction: Trends in the Prevalence of Tuberous Sclerosis Complex Manifestations: An Epidemiological Study of 166 Japanese Patients

**DOI:** 10.1371/annotation/c511ee3c-f91a-4cfe-bad7-6ef58579717e

**Published:** 2013-12-20

**Authors:** Mari Wataya-Kaneda, Mari Tanaka, Toshimitsu Hamasaki, Ichiro Katayama

Figure 4 is incorrect. Please view the correct Figure 4 here: http://plosone.org/corrections/pone.0063910.g004.cn.tif

